# Preliminary study on the active substances and cellular pathways of lactic acid bacteria for colorectal cancer treatment

**DOI:** 10.7150/jca.94530

**Published:** 2024-07-16

**Authors:** Si-Hui Zhao, Shu-Ming Zhang, Jin-Wei Yang, Chen-Jian Liu, Xue-Qin Zeng, Yuan-Lian Zhang, Si-Qian Chen, Zhi-Min Zhao, Yun-Xin Xia, Xiao-Ran Li, Yun Shang

**Affiliations:** 1 Second Department of General Surgery, First People' s Hospital of Yunnan Province, Kunming 650032, Yunnan, China.; 2 Second Department of General Surgery, Affiliated Hospital of Kunming University of Science and Technology, Kunming 650032, Yunnan, China.; 3 Faculty of Life Science and Technology, Kunming University of Science and Technology, Kunming 650500, Yunnan, China.; 4 Institute of Neuroscience, Kunming Medical University, Kunming 650500, Yunnan, China.

**Keywords:** CRC, *Lb. plantarum* AY01, 2'-Deoxyinosine

## Abstract

Colorectal cancer (CRC) is a common malignant tumor and is one of the three most common cancers worldwide. Traditional surgical treatment, supplemented by chemotherapy and radiotherapy, has obvious side effects on patients. Immunotherapy may lead to some unpredictable complications. Low introduction rate and high cost are some of the problems of gene therapy, so finding a safe, reliable and least toxic treatment method became the main research direction for this study. Lactic acid bacteria and their metabolites are widely used in functional foods or as adjuvant therapies for various diseases because they are safe to eat and have no adverse reactions. Research has shown that lactic acid bacteria and their metabolites play an auxiliary therapeutic role in colorectal cancer mainly by improving the intestinal flora composition, inhibiting the growth of pathogenic bacteria and inhibiting the proliferation of cancer cells. It is now widely believed that the substances that probiotics such as lactic acid bacteria exert anti-cancer effects are mainly secondary metabolites such as butyric acid. *Lb. plantarum* AY01 isolated from fermented food has good anti-cancer ability, and its main anti-cancer substance is 2'-deoxyinosine. Through flow cytometry detection, it was found that *Lb. plantarum* AY01 can block cell proliferation in the S phase. In addition, *Lb. plantarum* AY01 culture reduces the sensitivity of mice to colitis-associated CRC induced by azoxymethane (AOM)/dextran sulfate sodium salt (DSS) and exhibits the occurrence and promotion of tumors. According to transcriptome analysis, *Lb. plantarum* AY01 may induce apoptosis of colorectal cancer cells by activating the p38 MAPK pathway. This experiment provided possibilities for the treatment of CRC.

## Introduction

Colorectal cancer (CRC) is one of the most common cancer types, with the third highest incidence rate and the fourth highest mortality rate of all cancer types worldwide 1. Each year, 1.3 million new CRC cases occur, threatening 694,000 lives worldwide 2. The occurrence and development of CRC are associated with inheritance, the environment, daily diets and other factors 3-5. However, only 20-25% of cases are related to genetic factors 6. Research has shown that improper eating habits, including the consumption of fat, alcohol and red meat, are the most important risk factors for CRC 7, 8. An unhealthy intestinal state is usually accompanied by dysbacteriosis and disturbance of normal intestinal flora 9. The intestinal microbial community composition of CRC patients changed significantly compared with that of healthy people 10-13. Previous investigations have suggested that probiotics can improve the intestinal flora. Probiotics can alter the intestinal fora by reducing intestinal pH, competing for nutrition and secreting antibacterial compounds 14-16. Clavijo et al. 17 reviewed the role of microbiota in the gastrointestinal tract of broilers and discussed the important role of probiotics in the control of pathogens. Le Leu et al. 18 examined whether the symbiotic combination of *Bifidobacterium lactis* (*B. lactis*) and resistant starch could change gut microbial composition. We found that yogurt fermented with different probiotics significantly changed the intestinal flora composition of 144 mice with slow transit constipation and improved defecation time and intestinal health 19.

At present, chemotherapy, radiotherapy and surgery are still the main methods for the treatment of CRC; however, their clinical utility remains hampered by their toxicity and side effects 20. Surgery is the main treatment for CRC. However, the possibility of postsurgical trauma, decreased intestinal mucosal barrier function, intestinal flora disturbance, increased systemic inflammation, decreased immune function, and the risk of postoperative infection is also greater 21. Therefore, enhancing the immune response and improving the gut microbiota are effective strategies to prevent CRC 22. According to data from the World Health Organization and the Food and Agriculture Organization of the United Nations, a proper dose of probiotics has a beneficial effect on human health. Probiotics are retained in the body without any side effects, which has been recognized by the public 23. Studies have shown that probiotics are closely connected with various health benefits, including the conditioning of the intestinal microflora, suppression of excess allergic responses and tumor suppressive effects 24, 25. Dos Reis et al. 26 reviewed that treatment with probiotics for CRC could improve the composition of the intestinal microbial community, change the metabolic activity of intestinal microorganisms, break down carcinogens and reduce their carcinogenic activity, produce compounds with anticancer activity, enhance autoimmunity, strengthen the intestinal barrier, change host physiology, inhibit cancer cell proliferation and induce cancer cell apoptosis. Zhang et al. 27 used probiotics in postoperative CRC patients and found that the recurrence rate of the experimental group using probiotics was 10%, while that of the control group without probiotics was 33.3%. Mohania et al. 28 showed that the proliferation of cancer cells could be inhibited either by probiotics alone or in combination with cancer drugs in CRC mice. Many studies have confirmed that lactic acid bacteria (LAB) play an important role in preventing postoperative abdominal infection or intestinal complications 29. Some researchers have attempted to find solutions from feces, but gut microbiota may pose potential risks, while probiotics from food sources are naturally safe. Ishikawa et al. 30 discovered that the occurrence rate of tumors with a grade of moderate atypia or higher was significantly lower in the group administered *Lactiplantibacillus casei* (*Lb. casei*). Konishi et al. 31 showed that the culture supernatant of *Lb. casei* ATCC334 has a strong tumor-suppressive effect on colon cancer cells. Sadeghi et al. 32 found that heat-inactivated *Lactiplantibacillus plantarum* (*Lb. plantarum* A7), *Lactiplantibacillus rhamnosus* GG (*Lb. rhamnosus* GG) and aseptic cell culture supernatant had good inhibitory effects on Caco-2 and HT-29 cells.

Studies have shown that LAB, especially *Lb. plantarum*, and their metabolites can inhibit CRC. Hu et al. 33 showed that oral administration of *Lb. plantarum* in BALB/c mice inhibited the growth of CT26 cells and prolonged the survival time of tumor-bearing mice by increasing the effector function of CD8+ and infiltration of natural killer (NK) cells into tumor tissues, upregulating IFN-γ and promoting Th1-type CD4+ differentiation. Faghfoori et al. 34 concluded that the culture supernatant of *Lb. plantarum* showed significant cytotoxic effects on Caco-2 cancer cells. Mushtaq et al. 35 observed that the water-soluble extract (WSE) of kalari cheese with added *Lb. plantarum* exhibited profound cell proliferation inhibition against MCF-7, HCT-116, IMR-32, and HEK-T cancer lines. These studies have shown that host microbial interactions bring health benefits to the host by regulating specific molecular mechanisms of symbiotic bacteria and probiotics. However, the antitumorigenic molecules produced by commensal bacteria and probiotics have not been identified. However, the antitumorigenic molecules involved remain unclear.

Therefore, this study screened ten strains of lactic acid bacteria isolated from fermented foods to test their resistance to colorectal cancer to find the most effective lactic acid bacteria for colorectal cancer treatment. Transcriptome analysis of colorectal cancer cells infected with freeze-dried powder of selected lactic acid bacteria was performed. At the same time, the lactic acid bacteria were screened for anti-colorectal cancer substances to obtain lactic acid bacteria with good anti-colorectal cancer ability and clarify the underlying mechanism.

## Materials and Methods

### Strains and LAB Culture

Ten strains of lactic acid bacteria were isolated from traditional fermented foods in Yunnan Province (Table 1).

Ten strains were activated in 5 mL Man-Rogosa-Sharpe (MRS). The strains were cultured at 37 ℃ for 24 hours. Ten strains were inoculated into 100 mL of MRS medium for expansion cultivation (inoculation concentration was 10^5^ CFU/mL, cultivation time was 72 h). After 72 hours of culture, the LAB culture solution was centrifuged for 15 min at 10 000 g and 4 °C. After centrifugation, the supernatant (metabolite of LAB) was collected, and the precipitate was discarded. The culture supernatant of LAB was frozen to lyophilized powder using a laboratory vacuum freeze-drying machine SJIN-10 N (Ningbo Shuangjia Instrument Co., Ltd, China). The lyophilized powder was dissolved in RPMI 1640 (BI, Israel) medium without fetal bovine serum (FBS, BI, Israel) at a concentration of 0.5 g/mL. After dissolution, the solution was filtered through a 0.22 μm filter (Biosharp, China) and then stored in the refrigerator at -20 °C.

### Cell Assay

#### Cell Culture

The human colon cancer cell lines HT-29, HCT-116, Caco-2, and SW-620 were all purchased from Kunming Institute of Zoology, Chinese Academy of Sciences. Cell lines were grown in high-glucose Roswell Park Memorial Institute (RPMI) 1640 (HT-29) or Dulbecco's Modified Eagle's Medium (DMEM, BI, Israel)) (HCT-116, Caco-2 and SW-620) supplemented with 10% (v*v-1) FBS, 2 mM L-glutamine, 50 IU/mL penicillin (BI, Israel) and 50 μg/mL streptomycin (BI, Israel) in a humidified atmosphere containing 5% CO_2_.

#### MTT Assay

Cells were plated in 96-well plates at a density of 10^5^ cells/well. The cells were completely attached (approximately 12 h), and 100 μL of lyophilized powder solution of LAB culture supernatant at different concentrations was added (concentrations of 250 mg/mL, 125 mg/mL, 10 mg/mL, 5 mg/mL, and 1 mg/mL). Six biological replicates were set at each concentration. Serum-free RPMI 1640 medium of the same volume was added as a negative control. Cell growth was determined using the 3-(4,5-methylthiazol-2-yl)-2,5-diphenyltetrazolium bromide (MTT, BBI, China) colorimetric growth assay. Every 12 hours, cell growth was determined by adding MTT solution (125 μg/well) and incubating for 4 h. Cellular MTT was resolved with dimethyl sulfoxide (DMSO, Sangon Biotech, China) and measured at 540 nm. *Lb. plantarum* AY01 and *Lb. fermentum* JSL8-2 were selected for further experiments. Lyophilized powder of *Lb. plantarum* AY01 and *Lb. fermentum* JSL8-2 culture supernatant were diluted to 1 mg/mL, 2 mg/mL, 4 mg/mL, 8 mg/mL, 16 mg/mL, 32 mg/mL, 64 mg/mL, and 128 mg/mL. In this study, 5-Fluorouracil (5-FU, Sangon Biotech, China) and RPMI 1640 medium (without serum) were used as positive and negative controls, respectively. Six biological replicates were set at each concentration. Then, 100 μL of different culture supernatant solutions, RPMI 1640 medium (without serum) and 5-FU (10 μg/mL) were used to treat HT-29, Caco-2, HCT-116 and SW-620 cells. As soon as the lyophilized powder solution was added, the HT-29 cells were cultured in a 5% CO2 incubator at 37 °C for 24 h, 36 h, 48 h, and 60 h, and the Caco-2, HCT-116 and SW-620 cells were cultured in a 5% CO2 incubator at 37 °C for 48 h. The MTT assay was used to determine the survival rate of all cancer cells.

#### Flow Cytometry Detection

HT-29 cells in 1 mL (10^6^/mL) were added to 6-well plates and then cultured at 37 °C in a 5% CO_2_ incubator. After the cells were completely attached (approximately 12 h), 1 mL of lyophilized powder solution of *Lb. plantarum* AY01 and *Lb. fermentum* JSL8-2 culture supernatant were set at different concentrations. The final concentrations of lyophilized powder were 16 mg/mL, 32 mg/mL, and 64 mg/mL. As soon as the lyophilized powder solution was added, the HT-29 cells were cultured in a 5% CO_2_ incubator at 37 °C for 24 h and 48 h.

After cell culture, the cells were centrifuged at 10 000 g and 4 °C for 5 min. The supernatants were discarded, and the cells were collected. The cells were washed three times with precooled sterile PBS and centrifuged at 10 000 g and 4 °C for 5 min. Then, 1 mL 4',6-diamidino-2-phenylindole (DAPI, BBI, China) dye was added and mixed for detection by flow cytometry (Partec GmbH, Germany). The detection optical channel was FL-9. After detection, the data were analyzed by ModFit software.

#### Cell Apoptosis Detection

The cells were treated according to the above methods. The TransDetectTM Annexin V-FITC/PI Cell Apoptosis Detection Kit (TransGen Biotech, China) was used for detection: 1*Annexin V Binding Buffer was added into the cells to resuspend the cells, Annexin V-FITC and PI were added at 5 μL each and mixed gently, cell mixtures were placed at room temperature (20-25 °C) and reacted for 15 min without light, precooled 1*Annexin V Binding Buffer was added at 400 μL, mixed gently, and the samples were placed on ice without light. Flow cytometry was used to detect cell apoptosis within one hour. The detection channels were FL-1 and FL-3. In addition, three groups of control samples were set up: 1) negative control cells without any dye; 2) apoptotic-positive cells, single-stained Annexin V (without PI); and 3) apoptotic-positive cells, single-stained PI (without Annexin V).

### Animal Modeling

Male Kunming mice were purchased from Kunming Medical University and were raised in the Animal Room of Kunming University of Science and Technology. All mice were randomly divided into cages, each containing five mice. The mice were placed in a temperature controlled room (25 ± 2 °C) with humidity (50-70%) and low noise and isolated from all other ongoing animal experiments. The mice were kept under a 12:12 h light:dark cycle. All mice were given food and water ad libitum. All animal experiments were approved by the Animal Ethics Committee of the Academy of Kunming University of Science and Technology.

In the mouse experiment, 40 mice were randomly divided into three groups: control + vehicle (n = 10), azoxymethane (AOM)/dextran sulfate sodium salt (DSS) + vehicle (n = 15), and AOM/DSS + *Lb. plantarum* AY01 culture supernatant (AOM/DSS + *Lb. plantarum* AY01) (n = 15). At 2 weeks of age (baseline week 0), mice received either an intraperitoneal injection of the carcinogen 36 AOM (10 mg/kg) (Sigma, St. Louis, MO, America) diluted in physiological saline (PS) (AOM/DSS) or PS alone (Control). Mice receiving the AOM injection were subjected to three cycles of DSS-supplemented water (36-50 kDa, MP Biomedical, America) at final concentrations of 2.5%, 2% and 2% at weeks 1, 4 and 7, respectively. Each DSS cycle lasted for a 1 weeek period. AOM was administered on the first day, and then 500 μL of physiological saline or *Lb. plantarum* AY01 cell supernatant (128 mg/mL) was administered via oral gavage for 10 weeks to mice daily.

Calculation of the symptom score was performed as previously described 37, taking into account stool consistency and rectal bleeding. Briefly, fresh colonic evacuates were smeared onto “Hemoccult” tape to assess the severity of diarrhea and were tested with a developer (Beckman Coulter, Brea, CA) to assess rectal bleeding. Bleeding was scored as follows: no positive detection of blood (0), detection of blood but not grossly visible (2), and gross visibility of blood (4). Diarrhea was scored as solid cylinder (0), soft cylinder and easily spreadable (2), and noncylindrical or runny (4).

### Histological Analysis

Mice were euthanized by isoflurane (ChemeGen, America) at the end of 10 weeks. After sacrifice, the rectums and colons were harvested, flushed of feces and immediately fixed in 10% formalin (BBI, China) for 24 h for immunohistochemical and morphological analysis. Hematoxylin and eosin (BBI, China) staining of the rectum and colon was performed 38. Paraffin sections of rectal and colon tissue samples were made, and then immunohistochemical tests based on Ki-67 and PCNA were performed on these samples. Immunohistochemical staining of Ki-67 and PCNA was performed as previously described 39.

### Transcriptome Analysis of *Lb. plantarum* AY01 Anti-colorectal Cancer Cell (HT-29)

Cells with culture supernatant of *L. plantarum* AY01 treatment and control were collected by centrifugation. Total RNA was extracted using the TRIzol method according to the manufacturer's recommendations and assessed using an Agilent RNA 6000 Nano Kit on a Bioanalyzer 2100 (Agilent Technologies). Enriched mRNA using the Dynabeads® mRNA DIRECT™ Kit (Life Technologies) was used to construct mRNA-seq libraries using the KAPA Stranded mRNA-Seq Kit Illumina® platform according to the manufacturer's instructions.

The differentially expressed genes between the treatment and control were identified using the RUVSeq package (version 1.0.0, http://www.bioconductor.org). The DE genes had an average expression abundance of more than ten FKPM in either group with a false discovery rate less than 0.001 (FDR< 0.001) 40 and an absolute fold change (FC, treated/control) of more than three.

The hclust function of the stats package in R software (https://www.r-project.org/) was applied to perform hierarchical cluster analysis of DE genes between the treated cells and control cells. A heatmap for the DE genes was plotted using the heatmap.2 function in the gplots package.

The interaction network diagram of different genes was calculated by STRING 41, and the data with interaction coefficients greater than 0.7 were screened and imported into Cytoscape 42 to explore the network characteristics constructed by the whole transcriptome. The cell apoptosis process was obtained by GO enrichment using Clue GO 43 and Clue Pedia 44. Cytohubba 45 was used to calculate the core genes in the interaction network (MCC algorithm). GEPIA2 (http://gepia2.cancer-pku.cn/) was used to identify core genes.

### Crude Extraction and analysis of the Active Ingredients of *Lb. plantarum* AY01 in Anti-colorectal Cancer Cells

#### *Lb. plantarum* AY01 Supernatant Acquisition

The activated *Lb. plantarum* AY01 was inoculated into 10 L of fresh MRS liquid culture medium with a CFU of 10^6^/mL and allowed to ferment at 37 ℃ for 96 hours. Subsequently, the samples were centrifuged with a large refrigerated centrifuge at 8000 rpm/min for 5 minutes, and the culture supernatant was collected.

#### Separation of the Lb. plantarum AY01 Crude Extraction Product

The supernatant of *Lb. plantarum* AY01 was separated by an MCI column and eluted with different proportions of methanol solution (the concentrations of the eluent were MeOH, MeOH:H_2_O=3:7, MeOH:H_2_O=6:4, and H_2_O). The separated solutions were subjected to thin layer chromatography, and elution segments with the same chromogenic point positions were merged. The combined eluent was subjected to water bath rotary evaporation (55 °C) to obtain the crude extraction product.

#### Liquid Chromatography‒mass Spectrometry (LC‒MS) Analysis of Crude Extraction Products from *Lb. plantarum* AY01

The crude extraction product of *Lb. plantarum* AY01 was dissolved in anhydrous ethanol and treated with 0.22 μm membrane filtration (Biosharp, China). The crude extraction products were separated using an Agilent ZORBAX SB-C18 column (4.6 × 150 mm, 5 μm) in LC‒MS, and the relevant mobile phase conditions are shown in Table 2. LC‒MS results are displayed in the Dictionary of Natural Product, NPEdia Compounds Search and The Natural Products Atlas to identify possible compounds by comparison. The toxicity of possible compounds can be calculated using the Cramer rules in Toxtree software.

#### High-performance Liquid Chromatography (HPLC) Analysis

The 2'-deoxyinosine standard and supernatant of *Lb. plantarum* AY01 was analyzed using HPLC (Agilent Technologies Inc., Santa Clara, CA). The supernatant of *Lb. plantarum* AY01 was filtered through a 0.45 μm filter (Biosharp, China). The supernatant was injected into an Agilent ZORBAX SB-C18 column (4.6 × 150 mm, 5 μm). The supernatant of *Lb. plantarum* AY01 were monitored at 220-320 nm 46. The HPLC gradient is shown in the table 3.

### Statistical Analysis

Data from duplicate biological samples from the same time point were combined and expressed as the means and standard deviation (SD). The Mann-Whitney U test was used to compare groups when the variable did not show a normal distribution or did not exhibit equal variances. One-way ANOVA was used for statistical analysis for group comparisons. A p value of < 0.05 was considered significant. Analyses were performed using SigmaPlot 11.0 (Systat, Germany). One-way ANOVA was used for statistical analysis for group comparisons.

### Nucleotide Sequence Accession Numbers

The transcriptomic data were deposited in the Bioproject database under accession number PRJNA643903.

## Results

### Screening for LAB with Tumor-Suppressive Effects

**Figure 1:**
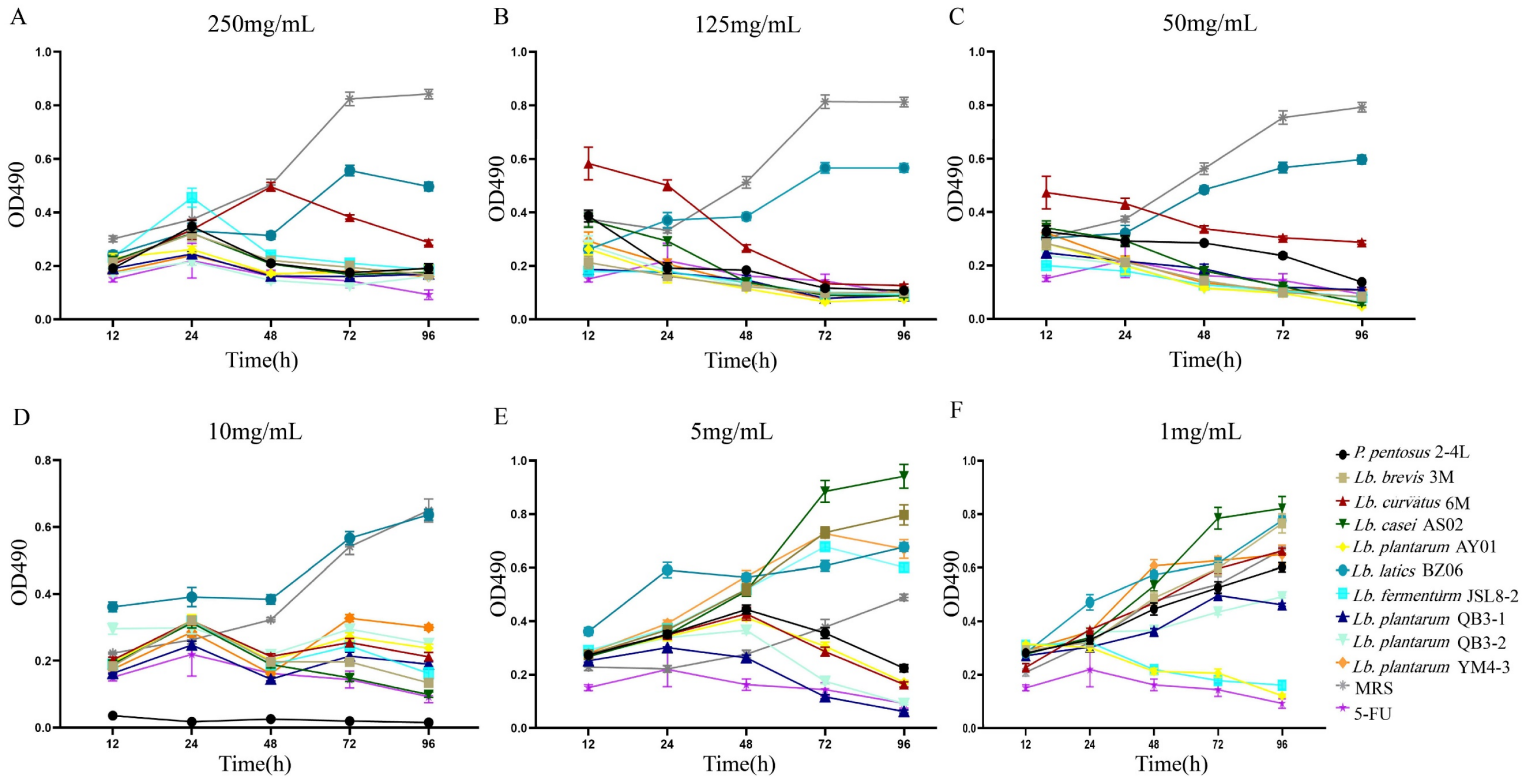
An MTT assay revealed that the numbers of HT-29 cells changed at different lyophilized powder concentrations, 250 mg/mL (**A**), 125 mg/mL (**B**), 50 mg/mL (**C**), 10 mg/mL (**D**), 5 mg/mL (**E**) and 1 mg/mL (**F**) of LAB culture supernatant. The error bars show the s.d. (n =6).

The lyophilized powder solutions of the culture supernatant of 10 strains of LAB showed different effects on HT-29 cells at different concentrations. Except for BZ06, the culture supernatants of the other nine strains of LAB showed good inhibitory effects on HT-29 cells at high concentrations (Figure 1A-D). With the decrease in the culture supernatant concentrations of nine strains of LAB, the inhibitory effect on HT-29 cells gradually decreased (Figure 1A-F). When the final concentrations were 1 mg/mL, only *Lb. plantarum* AY01 and *Lb. fermentum* JSL8-2 inhibited the proliferation of HT-29 cells (Figure 1F). Therefore, the preliminary screening results showed that *Lb. plantarum* AY01 and *Lb. fermentum* JSL8-2 had better inhibitory effects on HT-29 cells.

**Figure 2:**
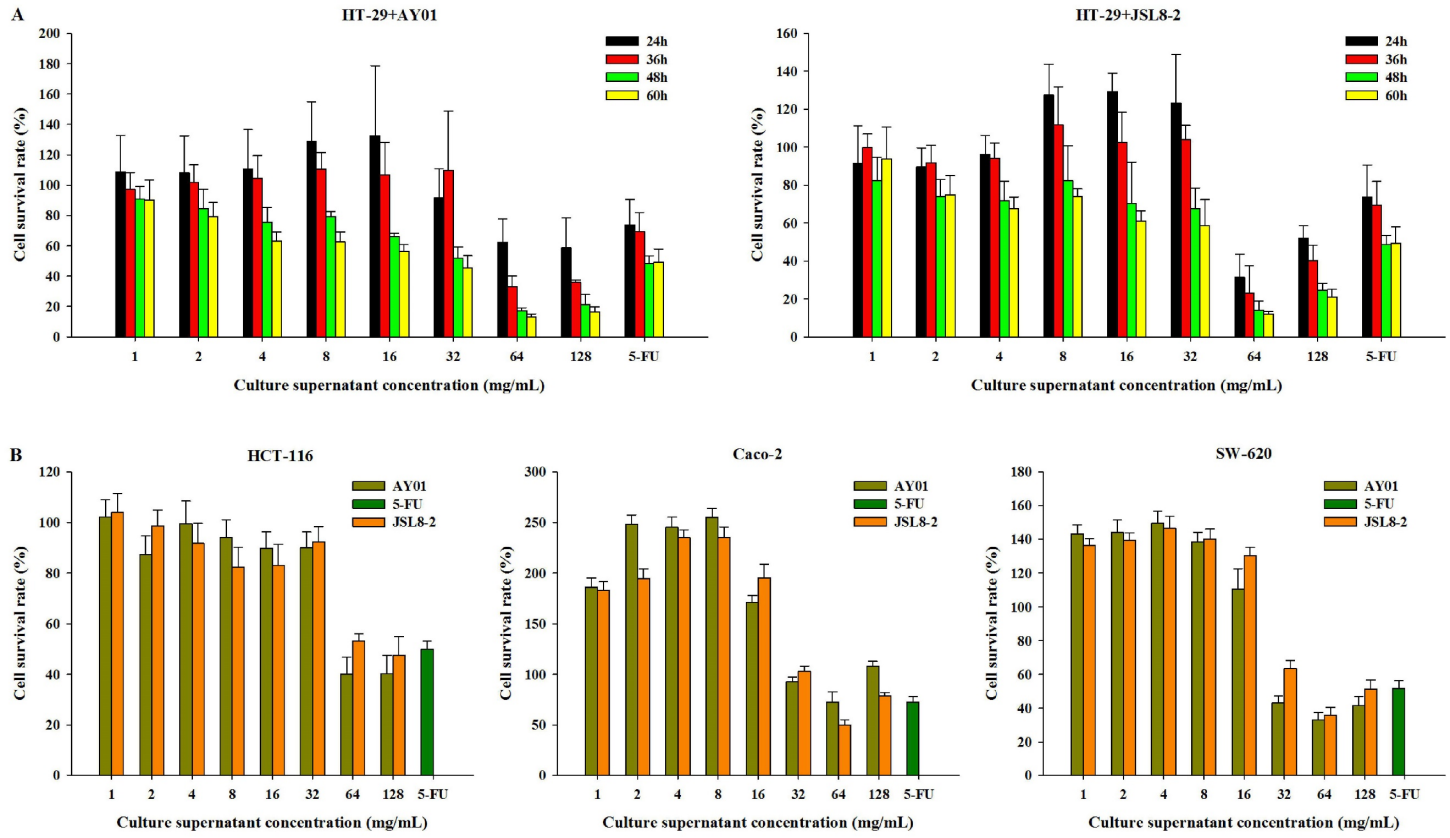
The survival rate of cancer cells detected by the MTT assay. (**A**) The survival rate of HT-29 cells treated with different culture supernatant concentrations of AY01 and JSL8-2 at different time. (**B**) The survival rate of HCT-116, Caco-2 and SW-620 cells treated with different culture supernatant concentrations of AY01 and JSL8-2 at 48 h. The error bars show the s.d. (n =6)

To determine the inhibitory effect of *Lb. plantarum* AY01 and *Lb. fermentum* JSL8-2 on colon cancer cells, HT-29 cells were treated with different lyophilized powder concentrations of *Lb. plantarum* AY01 and *Lb. fermentum* JSL8-2 for different times. The higher the lyophilized powder concentration of *Lb. plantarum* AY01 and *Lb. fermentum* JSL8-2 was, the lower the cell survival rate (Figure 2A), indicating a better inhibition effect of *Lb. plantarum* AY01 and *Lb. fermentum* JSL8-2 on HT-29 cells. At concentrations of 64 mg/mL and 128 mg/mL, *Lb. plantarum* AY01 and *Lb. fermentum* JSL8-2 had a good inhibitory effect (especially at 64 mg/mL). In addition, HT-29 cells treated with low concentrations of lyophilized powder solution grew faster than the blank control when the incubation time was less than 48 h, and the cell survival rate exceeded 100%. The treatment of HT-29 cells with two strains of bacteria showed the best effect at 48 h, so the final cultivation time of the subsequent experiment was set at 48 h. Lyophilized powders of *Lb. plantarum* AY01 and *Lb. fermentum* JSL8-2 also suppressed the growth of HCT-116, Caco-2 and SW-620 cells (Figure 2B). These data indicated that the secreted molecules of *Lb. plantarum* AY01 and *Lb. fermentum* JSL8-2 inhibited the growth of colon cancer cells.

### The Cell Cycle and Apoptosis of HT-29 Cells

**Figure 3:**
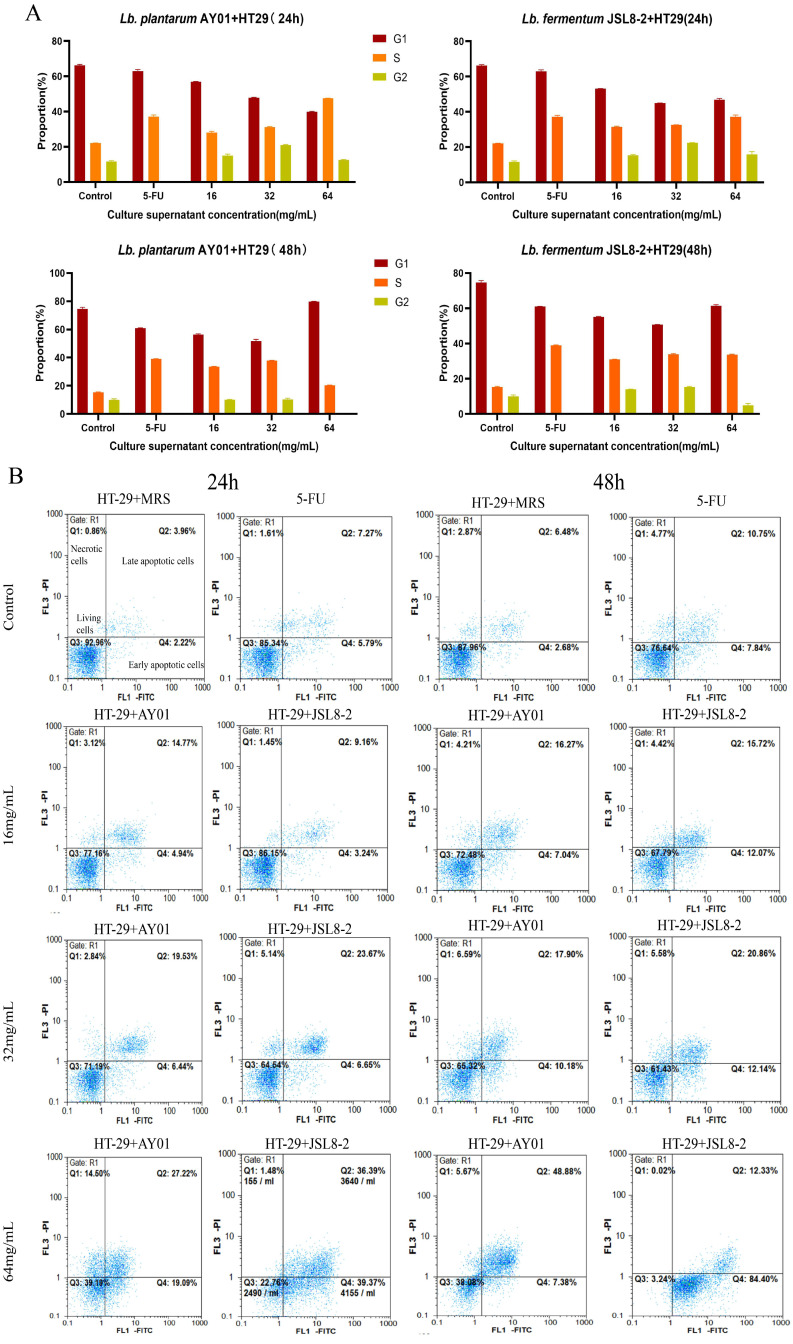
The cell cycle and apoptosis of HT-29 cells by flow cytometry. (**A**) The cell cycle of HT-29 cells treated with different concentrations of AY01, JSL8-2 culture supernatant and MRS (negative controls) and 5-FU (positive controls). (**B**) Apoptosis results of HT-29 cells treated with different concentrations of AY01, JSL8-2 culture supernatant and MRS (negative controls) and 5-FU (positive controls).

To investigate the inhibitory mechanism of *Lb. plantarum* AY01 and *Lb. fermentum* JSL8-2 on the growth of HT-29 cells, the cell cycle and apoptosis of HT-29 cells were detected by flow cytometry, MRS was used as a negative control (the cell cycle represented normal cells), and 10 μg/mL of 5-FU was used as a positive control. The cell cycle was divided into G1 phase, S phase, G2 phase and M phase. From the control group of MRS (24 h), two peaks were observed in the G1 phase and G2 phase, with proportions of 66.63% and 11.20%, respectively (Figure 3A). The 5-FU control group showed that the proportion of G2 phase had dropped to 0, and the S phase increased to 37.76%, indicating that 5-FU could block cells in S phase, which prevented cells from undergoing the complete cell cycle and the proliferation process was blocked (Figure 3A). Neither the concentration of *Lb. plantarum* AY01 nor *Lb. fermentum* JSL8-2 could cause HT-29 cell cycle arrest, and their cell cycle distribution was approximately the same as that of MRS, except that the ratios were slightly different (Figure 3A).

However, at a concentration of 64 mg/mL for 48 h, the cell cycle of HT-29 cells could block cells in S phase treated with *Lb. plantarum* AY01 and decreased the ratio of cells in G2 phase treated with *Lb. fermentum* JSL8-2 (Figure 3A), indicating that the culture supernatant of *Lb. plantarum* AY01 and *Lb. fermentum* JSL8-2 at 64 mg/mL could block the cell cycle of HT-29 cells. The proportion of S phase increased in the low concentration treatment group, and in the MTT experiment, the cell survival rate of the low concentration treatment group exceeded 100% when the incubation time was less than 48 hours. This indicates that short-term treatment with low concentrations of *Lb. plantarum* AY01 and *Lb. fermentum* JSL8-2 promotes the proliferation of HT-29, but long-term treatment still has an inhibitory effect on HT-29.

The results showed that *Lb. plantarum* AY01 and *Lb. fermentum* JSL8-2 induced apoptosis of HT-29 cells (Figure 3B). After treatment with MRS for 24 h, the proportion of living HT-29 cells was 92.96%, and the proportion of apoptotic cells was only 6.18%. The cells undergoing apoptosis were of normal growth, indicating that the cell status was good. When treated with 5-FU for 24 h, the percentage of HT-29 cells undergoing apoptosis increased from 6.18% to 13.06% compared with MRS, indicating that 5-FU can also induce apoptosis to some extent. After treatment of HT-29 cells with *Lb. plantarum* AY01 and* Lb. fermentum* JSL8-2 at concentrations of 16 mg/mL, 32 mg/mL and 64 mg/mL for 24 h, the apoptotic rates were 19.71% and 12.40%, 30.32% and 25.97%, 75.76% and 46.31%, respectively, and the inhibition rate was significantly higher than that of MRS. After treatment with MRS for 48 h, the apoptotic rate reached 9.16%, and after 48 h of treatment with 5-FU, the apoptotic rate was 18.59%, an increase of 5.53% compared with that at 24 h, indicating that the cells were naturally undergoing apoptosis under natural conditions with increasing culture time. After treatment with *Lb. plantarum* AY01 and *Lb. fermentum* JSL8-2 at concentrations of 16 mg/mL, 32 mg/mL and 64 mg/mL for 48 h, the apoptotic rate increased significantly to 27.79% and 23.31%, 33.00% and 28.08%, 96.73% and 56.26%, respectively. The induction effect was better as the concentration of the lyophilized powder solution increased. Obviously, as the treatment time increased, the effect of inducing apoptosis was also better, except for natural apoptosis. Notably, *Lb. plantarum* AY01 induced apoptosis of HT-29 cells significantly more than *Lb. fermentum* JSL8-2.

The results showed that the culture supernatant of *Lb. plantarum* AY01 and *Lb. fermentum* JSL8-2 inhibited the proliferation of HT-29 cells through cell cycle arrest and apoptosis. However, the culture supernatant of *Lb. plantarum* AY01 had a better inhibitory effect than *Lb. fermentum* JSL8-2. The best effect was achieved when HT-29 cells were treated with 64 mg mL-1 *Lb. plantarum* AY01 culture supernatant for 48 hours. The cell cycle of HT-29 cells was blocked in S phase, and the apoptotic rate reached 96.73%.

### The Decrease in the Sensitivity of Mice to AOM/DSS-induced Colitis-associated CRC through *Lb. plantarum* AY01 Culture Supernatant

**Figure 4:**
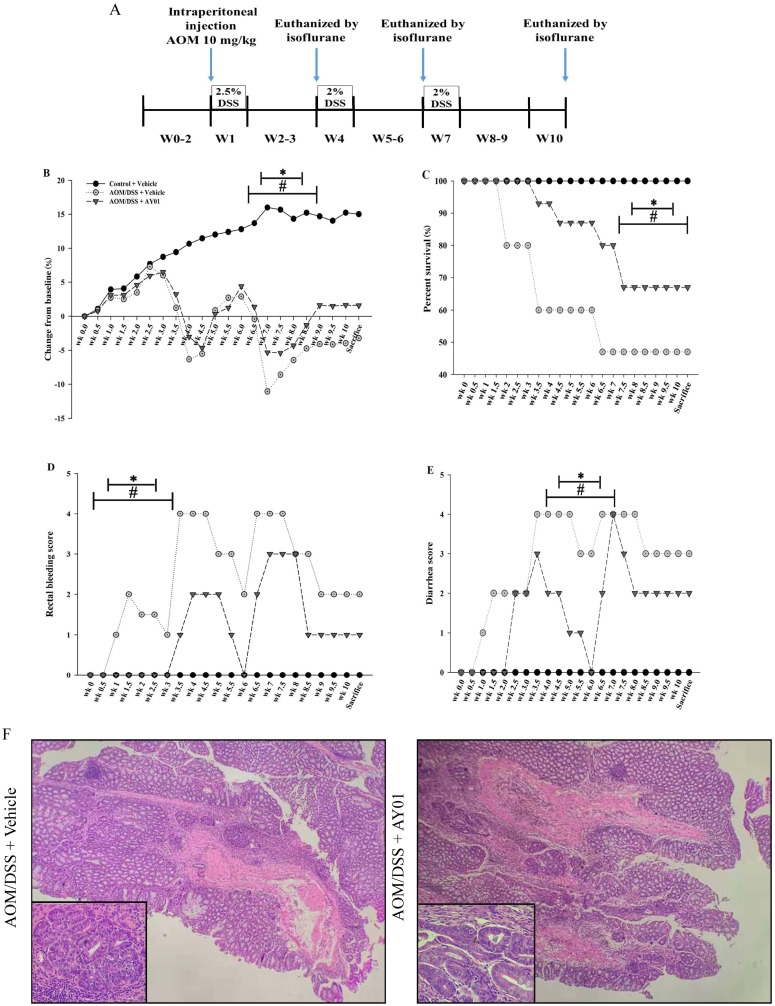
*Lb. plantarum* AY01 culture supernatant reduces sensitivity to AOM/DSS-induced colitis-associated CRC. (**A**) Animal experiment design. Mice were treated with AOM/DSS as Material and methods described. (**B**) Percent body weight change. The mean changes in body weight of the three groups mice were measured at the indicated time until week 10. (**C**) Survival curve. Their survival was monitored until week 10 after treatment with AOM. Rectal bleeding score (**D**) and diarrhea score (**E**). Bleeding and diarrhea score were scored as Material and methods described. # Indicates statistical significance (p < 0.05) for Control + Vehicle group vs. all groups, * indicates statistical signficiance (p < 0.05) for AOM/DSS + Vehicle vs. AOM/DSS + AY01, n=7-15/group. (**F**) H&E staining of colon tissue samples.

**Figure 5:**
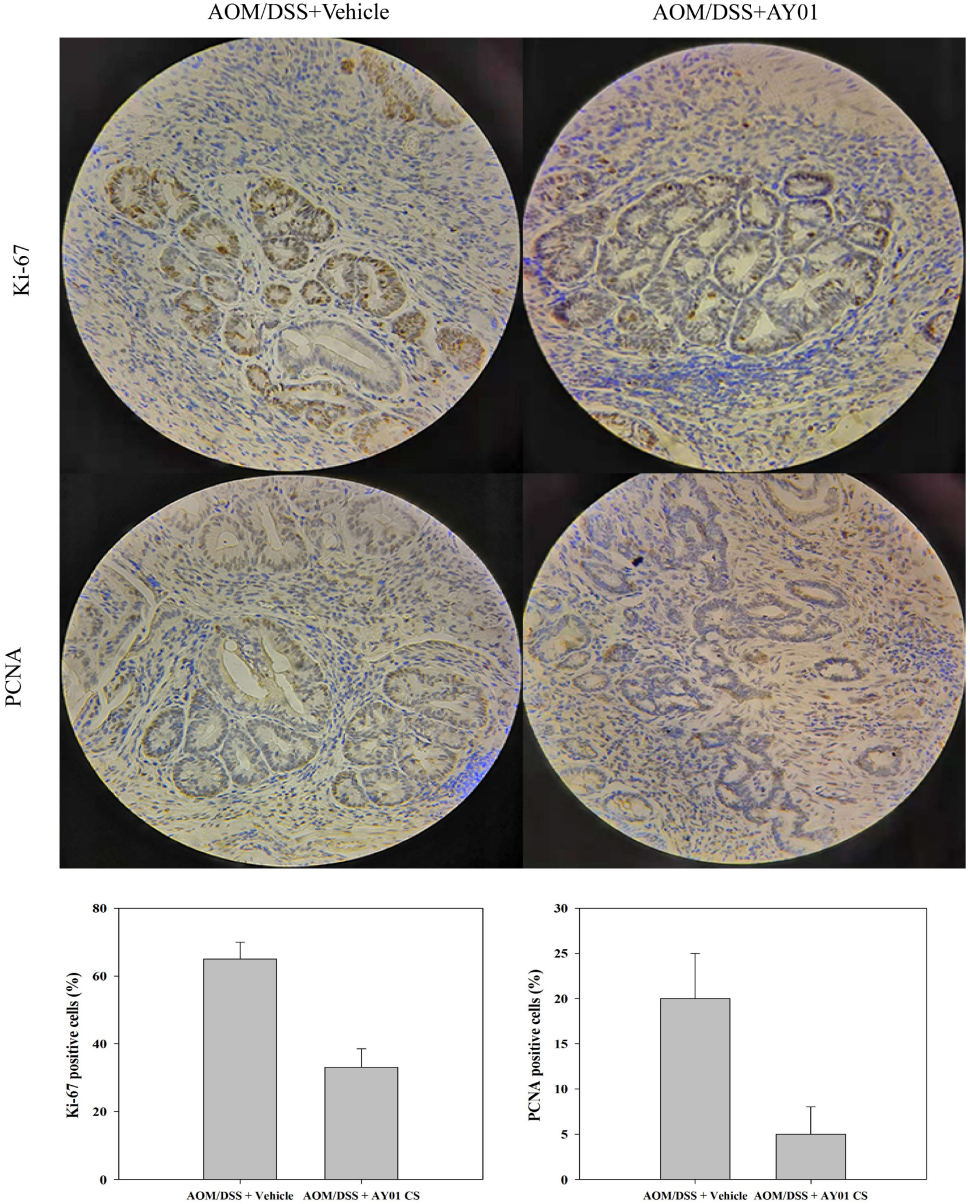
Ki-67 and PCNA immunohistochemistry staining (left panel) and percentage of Ki-67 and PCNA positive cells (right panel). * indicates statistical signficiance (p < 0.05) for AOM/DSS + Vehicle vs. AOM/DSS + AY01.

There was a main effect of *Lb. plantarum* AY01 culture supernatant on body weight loss, percent survival, rectal bleeding score and diarrhea score during DSS administration. In the second and third cycles, DSS caused noticeable weight loss and mortality in both AOM/DSS groups but that in the AOM/DSS + *Lb. plantarum* AY01 group was significantly lower than that in the AOM/DSS + Vehicle group (Figure 4B, C, P < 0.05), and by week 8, more than 50% of the mice in the AOM/DSS + Vehicle group had died. Consistent with this, a main effect of DSS on the severity of symptoms was seen during DSS administration, and the inflammation of AOM/DSS + *Lb. plantarum* AY01 group was alleviated. The AOM/DSS + Vehicle group exhibited greater symptom scores (including rectal bleeding score and diarrhea score) than the AOM/DSS + *Lb. plantarum* AY01 group (Figure 4D, E, P < 0.05). At the time of harvest after the third DSS cycle, H&E staining of colon tissue samples revealed highly differentiated adenoma in both the AOM/DSS + Vehicle and AOM/DSS + *Lb. plantarum* AY01 mice; remarkably, the mice in the AOM/DSS + Vehicle group had adenomas that infiltrated the muscle layer, while the mice in the AOM/DSS + *Lb. plantarum* AY01 group only had adenomas that infiltrated the submucosa (Figure 4F). We also counted Ki-67-stained cells and PCNA-stained cells and found a larger number of positive cells in AOM/DSS + Vehicle adenomas than in AOM/DSS + *Lb. plantarum* AY01 adenomas (Figure 5), suggesting that *Lb. plantarum* AY01 culture supernatant inhibits the proliferation of tumor cells.

These observations suggest that *Lb. plantarum* AY01 culture supernatant reduces the sensitivity of mice to AOM/DSS-induced colitis-associated CRC and then inhibits the occurrence and proliferation of tumors.

### Transcriptomic Analysis of HT29 Cells Treated with *Lb. plantarum* AY01 Supernatant

**Figure 6:**
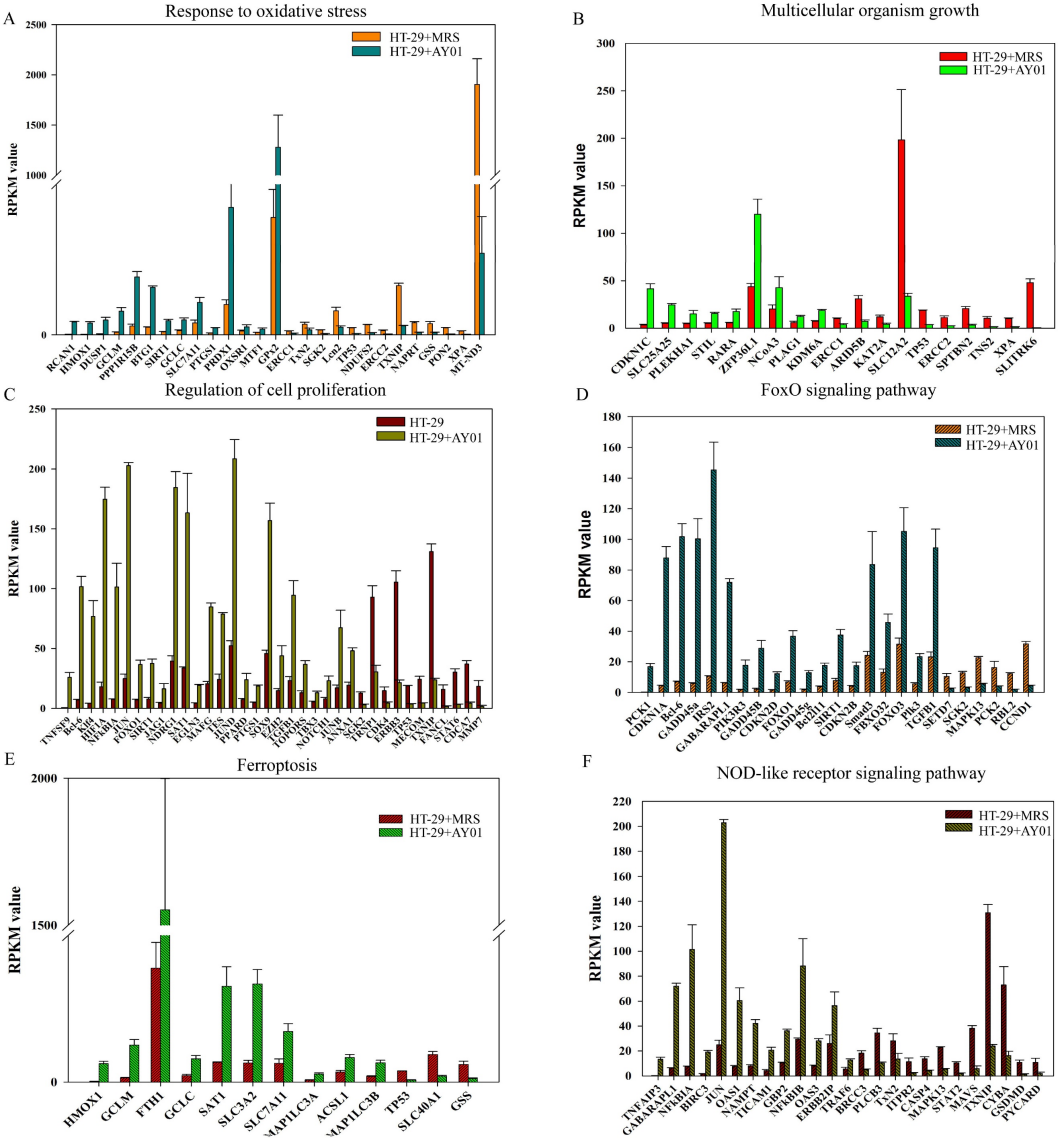
The difference of the gene expression levels in HT-29 cells with and without *Lb. plantarum* AY01 culture supernatant. The DEGs involved in response to oxidative stress (**A**), multicellular organism growth (**B**) and regulation of cell proliferation (**C**) by GO enrichment analyses. The DEGs involved in FoxO signaling pathway (**D**), Ferroptosis (**E**) and NOD-like receptor signaling pathway (**F**) by KEGG enrichment analyses.

To investigate the effect of the culture supernatant of *Lb. plantarum* AY01 on the gene expression of HT-29 cells, we examined the transcriptome analysis of HT-29 cells. Gene expression of HT-29 cells with and without *Lb. plantarum* AY01 culture supernatant were significantly different. The gene expression levels determined by the average RPKM values demonstrated that approximately 7000 of the total predicted genes were expressed, and there was a total of 1,215 significant differentially expressed genes. Among them, 628 genes were upregulated (HT-29 + *Lb. plantarum* AY01/HT-29), and 587 were downregulated. The results of GO enrichment analysis showed that the DEGs were mainly involved in the response to oxidative stress, multicellular organism growth and regulation of cell proliferation (Figure 6A-C). In response to oxidative stress, 14 genes, such as *RCAN1*, *HMOX1*, *DUSP1*, *GCLM*, and *PPP1R15B*, were upregulated, while 13 genes, such as *TP53*, *NDUFS2*, *ERCC2*, *TXNIP*, *NAPRT*, *GSS*, *PON2*, *XPA*, and *MT-ND3*, were downregulated (Figure 6A). Genes involved in multicellular organism growth were both up- and downregulated; upregulated genes included *CDKN1C*, *SLC25A25*, *PLEKHA1*, *STIL*, *RARA*, *ZFP36L1*, *NCOA3*, *PLAG1*, and *KDM6A*, and 10 genes were downregulated, including *TP53*, *ERCC2*, *SPTBN2*, *TNS2*, *XPA*, and *SLITRK6* (Figure 6B). A total of 25 upregulated genes, including *TNFSF9*, *Bcl-6*, *Klf4*, *HIF1A*, *NFkBIA*, and *JUN*, and 11 downregulated genes, including *STAT6*, *CDCA7* and *MMP7*, were involved in the regulation of cell proliferation (Figure 6C).

**Figure 7:**
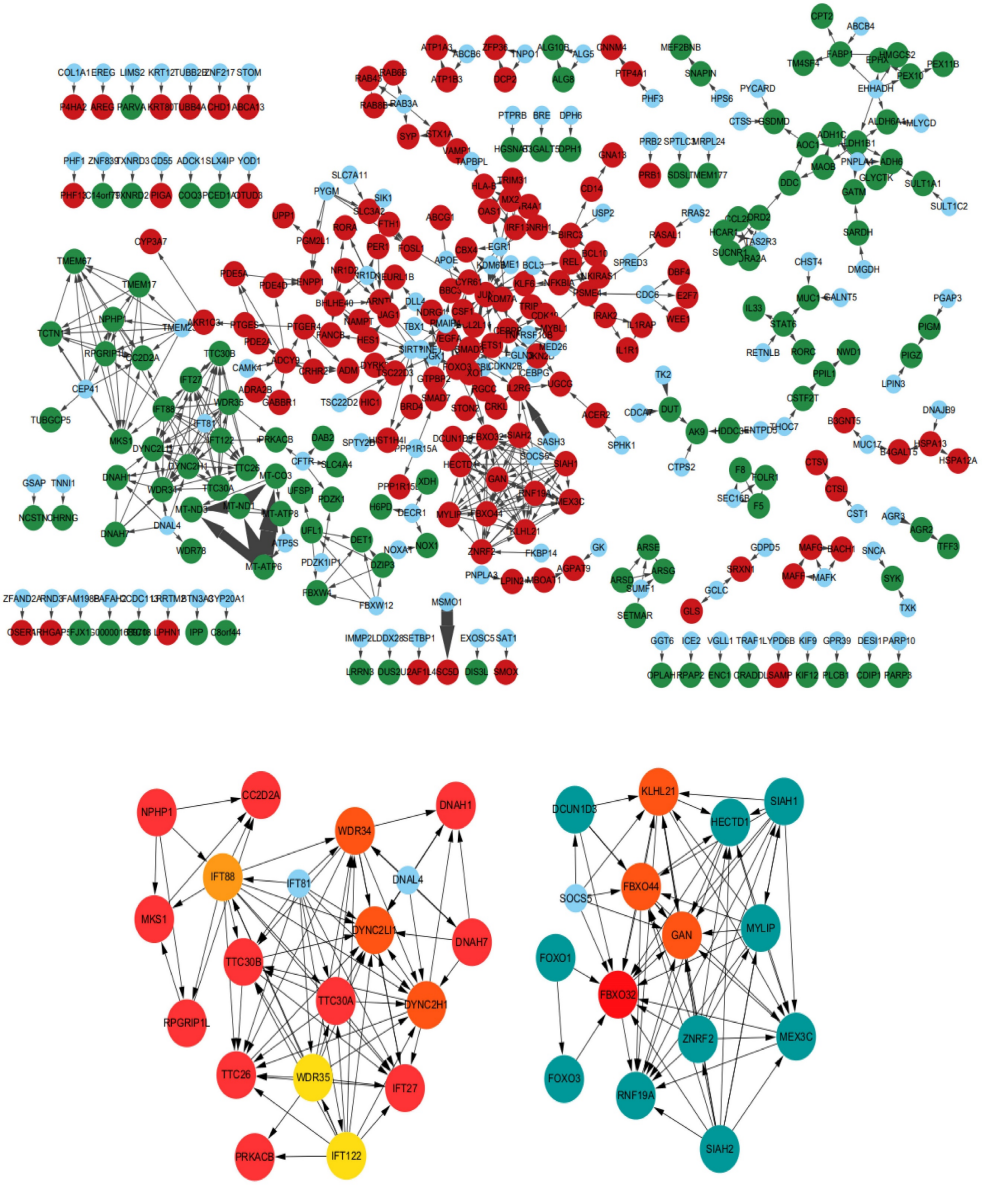
(**A**) Interaction network diagram of 1000 significantly different genes (500 significantly upregulated genes, 500 significantly downregulated genes, red indicating significant upregulation, green dots indicating significant downregulation, and blue dots indicating interaction related genes supplemented by STRING). (**B**) Ten core genes and their formation modules in differential gene interaction networks.

The KOBAS and DAVID databases were used for enrichment, and the list of main routes was obtained by sorting the P values from small to large (Table 4 and Table 5). It can be seen from the two tables that, at the transcription level, the anti-colorectal cancer effect of the *Lb. plantarum* AY01 strain mainly involves the FOXO pathway and p38 MAPK pathway. To explore the characteristics of the network constructed by the whole transcriptome, a network diagram of gene interactions and cell apoptosis was obtained by calculating and enriching genes. A gene interaction network diagram was calculated based on 1000 differentially expressed genes (Figure 7A). Two interaction networks composed of ten core genes, *FBXO32*, *WDR34*, *DYNC2H1*, *DYNC2LI1*, *KLHL21*, *FBXO44*, *GAN*, *IFT88*, *IFT122* and *WDR35*, were generated using the cytoHubba calculation (MCC algorithm) (Figure 7B).

**Figure 8:**
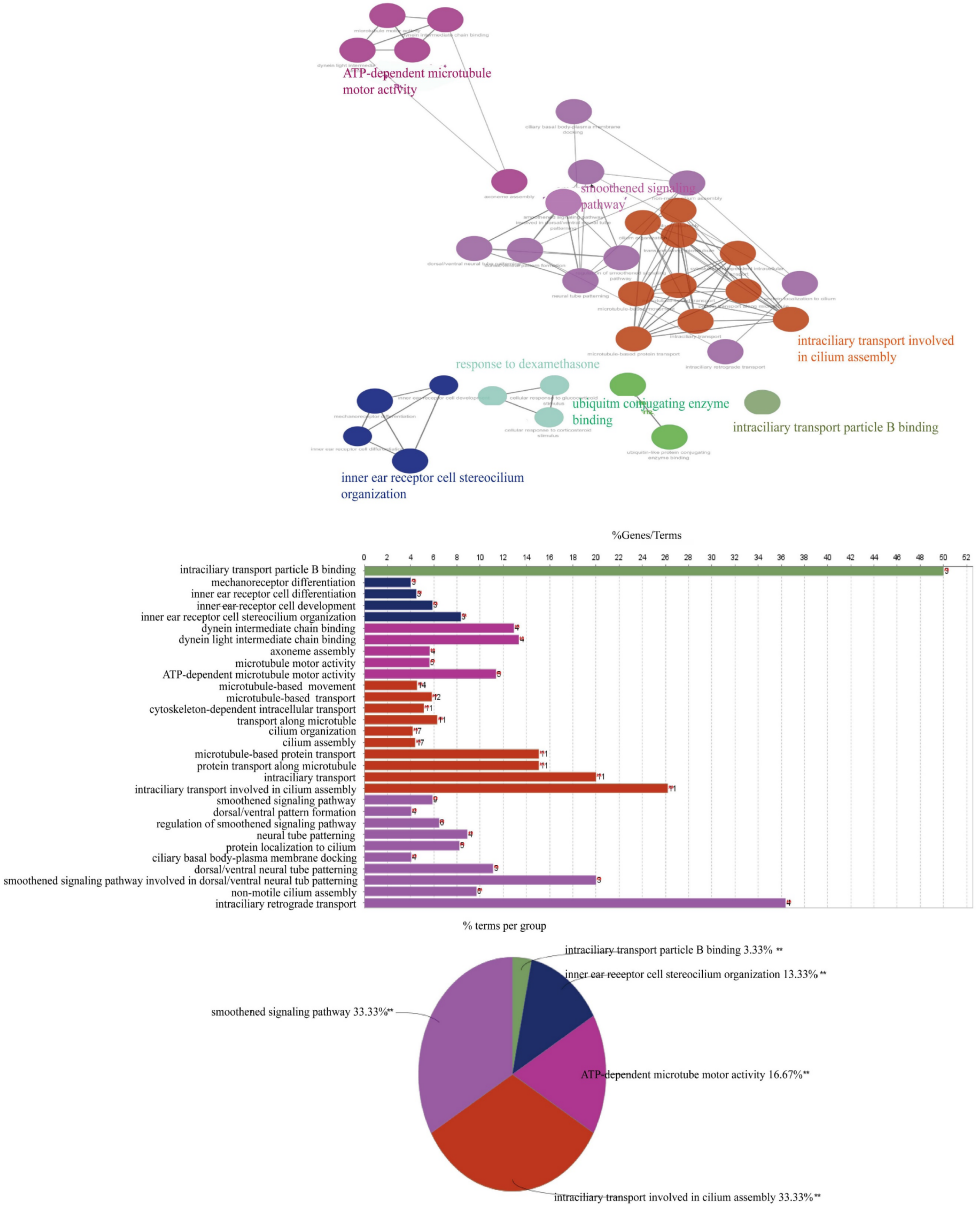
Molecular functions, biological processes, and immune annotations of the two networks in Figureure 6B. A total of 35 points and 112 edges were obtained, belonging to 5 groups, with the most important being ciliary assembly and ubiquitination processes.

According to the annotation of GO molecular function, biological process and immune process on the basis of these two subnets, 35 points and 112 edges were obtained, which belong to five groups, among which the most important are cilia assembly and ubiquitination process (Figure 8). According to previous studies, the above two subnetworks connect downstream of the p38 MAPK pathway through the FOXO pathway, and the p38 MAPK pathway is involved in the process of apoptosis. According to the analysis of the above transcriptome data, AYO1 can activate p38 MAPK to dredge collaterals and promote the apoptosis of cancer cells.

**Figure 9:**
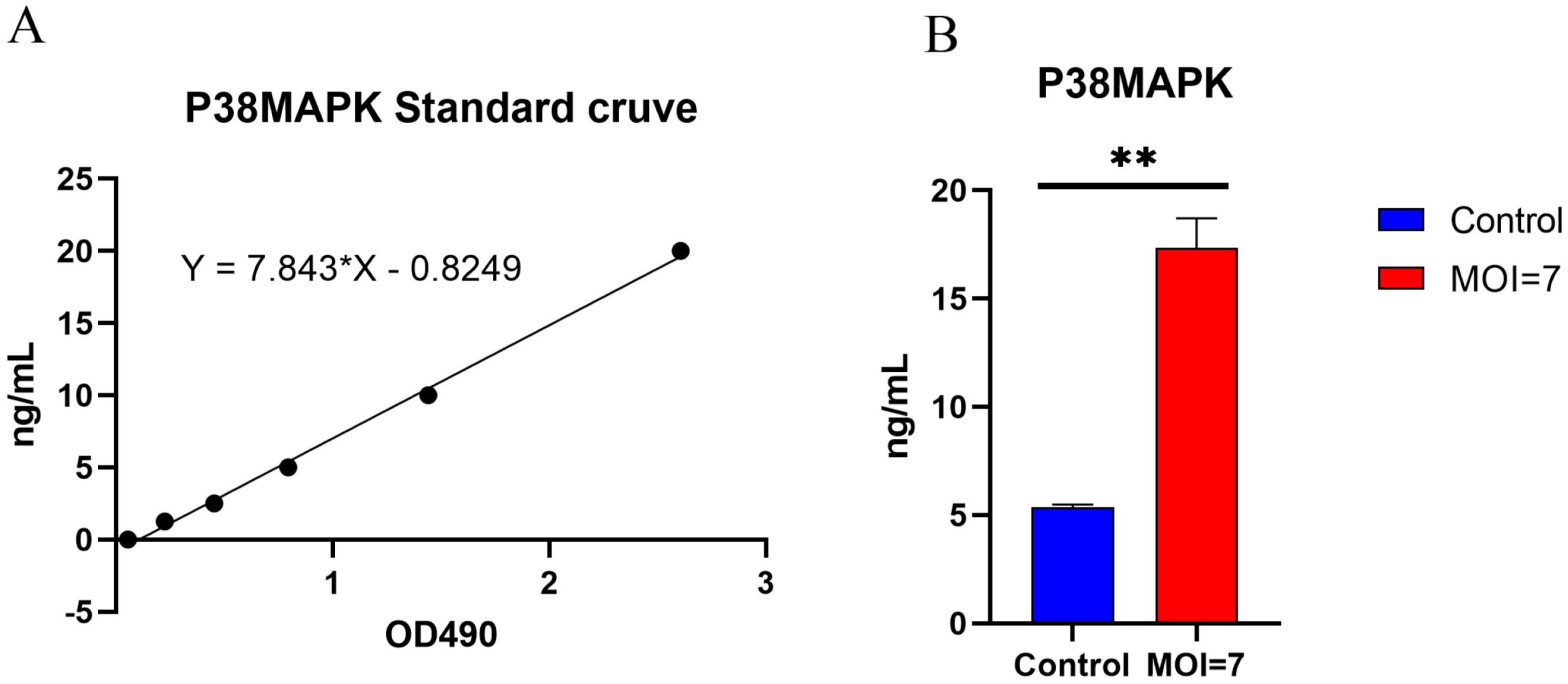
Expression of human p38 MAPK protein from HCT116 after co-cultured with AY01. (**A**) p38 MAPK ELISA standard curve. (**B**) the expression of p38 MAPK protein.

The p38 protein content in the co-cultured supernatant of AY01 and HCT116 was detected by Elisa human p38 MAPK assay kit (Figure 9). The p38 protein in the co-cultured supernatant of AY01 and HCT116 was significantly higher than that in the control group (HCT116 supernatant).

### 2'-Deoxyinosine is an Anti-colorectal Substance in *Lb. plantarum* AY01

**Figure 10:**
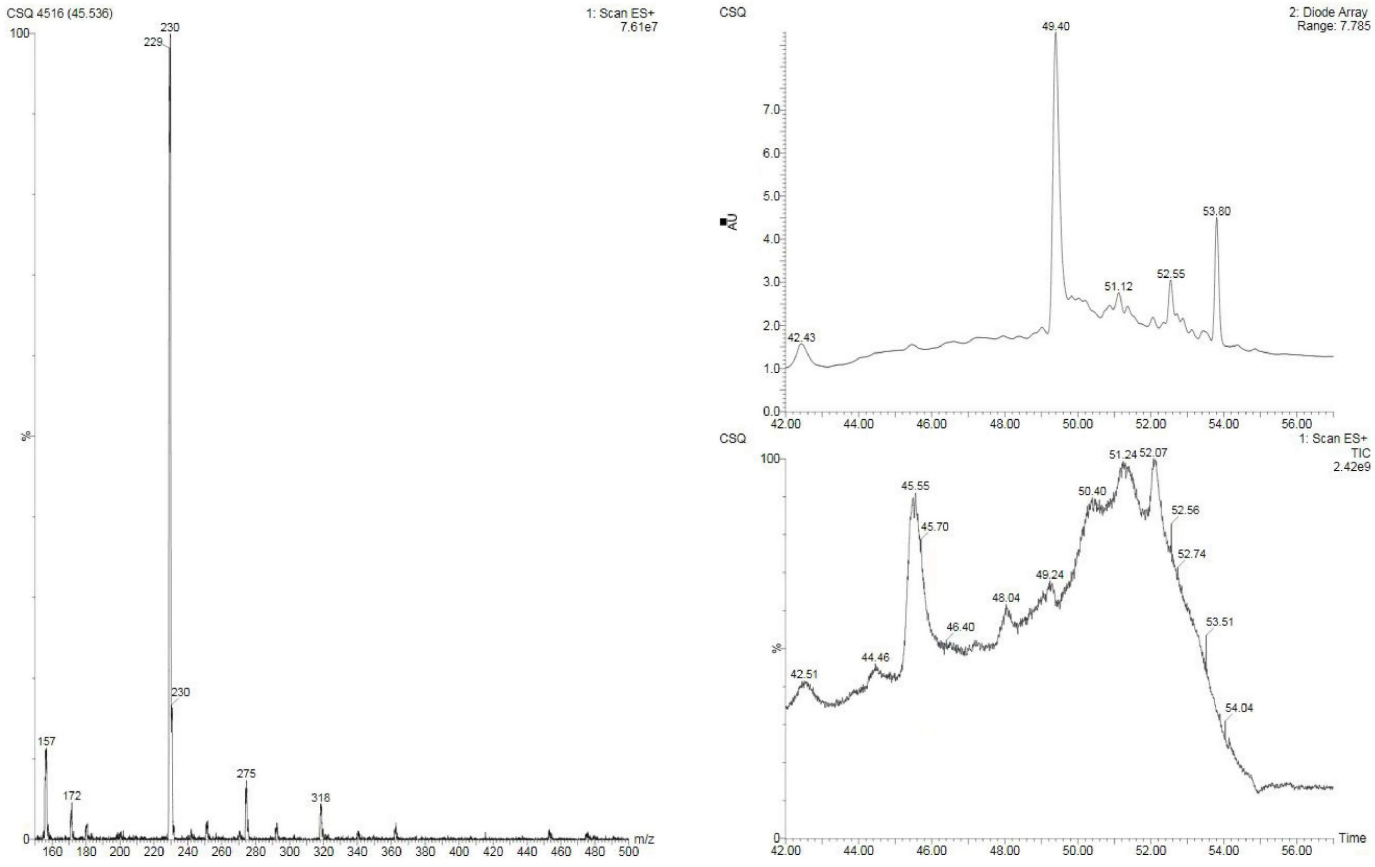
LC-MS Mass Spectrometry Results of AY01 Crude Extract.

**Figure 11:**
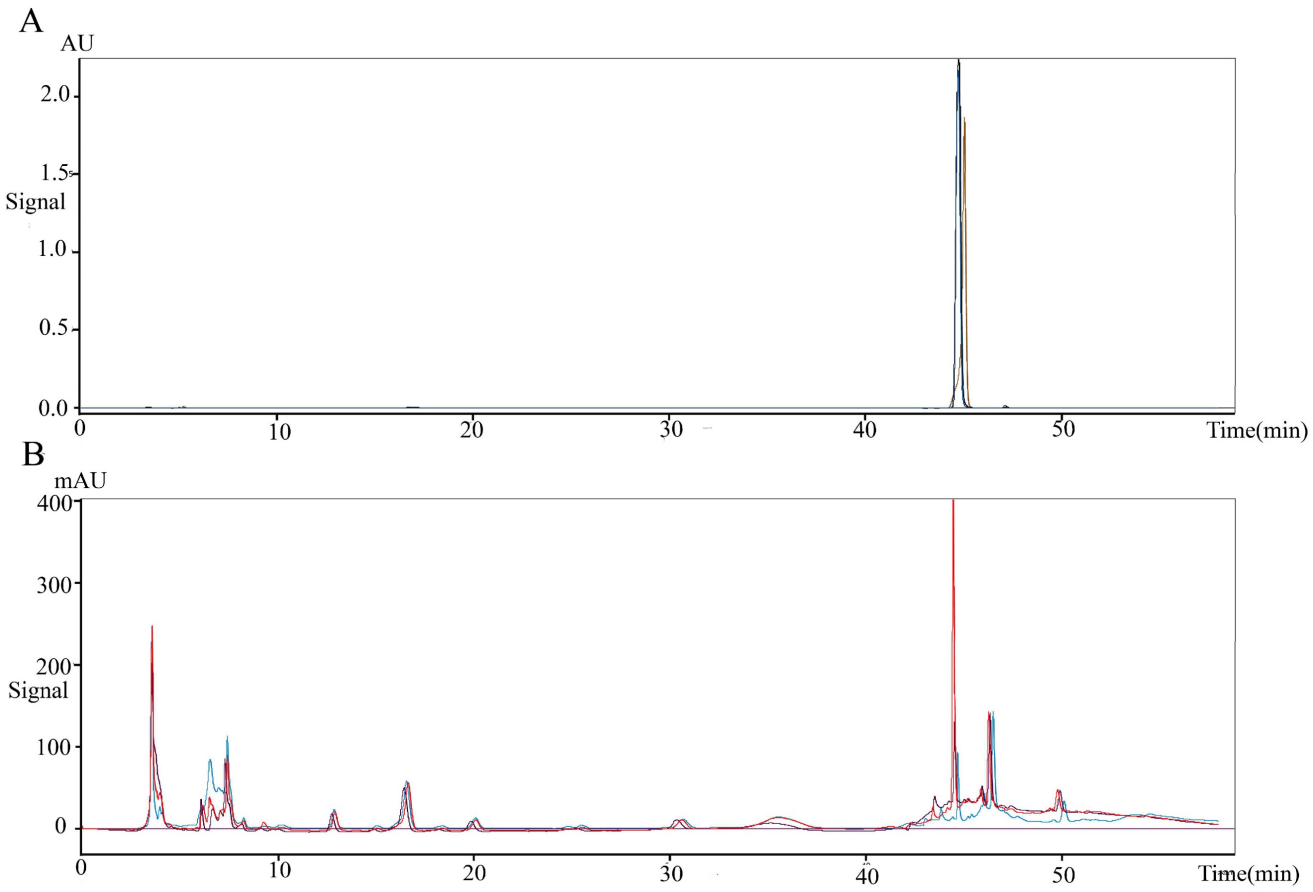
(**A**) 2'-Deoxyinosine standard peak appears between 40-48 minutes (repeat three times). (**B**) The peak appears in the supernatant of AY01 between 40-48 minutes (repeat three times).

To obtain the anti-colorectal cancer active substance, the fermentation products of *Lb. plantarum* AY01 were preliminarily isolated. The four fractions obtained for the first time were screened by the MTT method, and all of them had certain biological activity (pH was neutral), among which the activity of the fraction eluted with 100% methanol was the best (inhibition rate was 86.8%). Subsequently, the elution section of CH_2_Cl_2_: MeOH = 20:1 was found to have the best activity by further separation of this section. The substance segment with the highest concentration was subjected to subsequent separation. According to the molecular weight displayed by LC‒MS, the final separated product was compared with compounds with similar molecular weights (Figure 10). 2'-Deoxyinosine, found after comparison, can inhibit the growth of human cancer cell lines and is related to purine nucleoside phosphorylase (PNP) deficiency. The fermentation product of *Lb. plantarum* AY01 was detected by HPLC, and the peak time was consistent with that of the standard sample (Figure 11). Therefore, it is preliminarily considered that the active substance of *Lb. plantarum* AY01 against colorectal cancer is 2'-deoxyinosine.

## Discussion

In this study, *Lb. plantarum* AY01 and *Lb. fermentum* JSL8-2 were screened against human CRC cells by using the culture supernatant of LAB to test the inhibitory effect on CRC cell (HT-29, HCT-116, Caco-2 and SW-620) proliferation. Flow cytometry showed that *Lb. plantarum* AY01 and *Lb. fermentum* JSL8-2 could inhibit the proliferation of HT-29 cells by inducing apoptosis and blocking the cell cycle. However, *Lb. plantarum* AY01 had a more significant inhibitory effect than *Lb. fermentum* JSL8-2. *Lb. plantarum* AY01 significantly reduced the susceptibility of mice to AOM/DSS-induced colorectal cancer. Through transcriptome analysis, it was preliminarily judged that the anti-colorectal cancer effect of *Lb. plantarum* AY01 mainly involved the FOXO pathway and p38 MAPK pathway. The interaction network formed by ten core differentially expressed genes connects downstream of the p38 MAPK pathway through the FOXO pathway, which is involved in cell apoptosis. *Lb. plantarum* AY01 plays an important role in regulating cell cilia assembly and protein ubiquitination during the process of inducing apoptosis in colorectal cancer cells. The separation and extraction of anticancer substances from the supernatant of *Lb. plantarum* AY01 revealed that 2'-deoxyinosine was its anticancer substance. Moreover, 2'-deoxyinosine can block the growth of cancer cells in the S phase. Meanwhile, flow cytometry detected that the supernatant of *Lb. plantarum* AY01 also blocked the growth of colorectal cancer cells in the S phase.

Both *Lb. plantarum* and *Lb. fermentum* have been widely used in yogurt fermentation and kimchi production, and their safety and health promotion effects have been recognized. There have been studies using different concentrations of *Lb. fermentum* (180 mg/kg, 360 mg/kg and 720 mg/kg) to treat nude mice transplanted with the human colorectal cancer cell line HT-29 and it was found that the subcutaneous tumor inhibition rates in nude mice reached 63%, 79%, and 83%, respectively. This study found that the inhibition rate of *Lb. plantarum* AY01 culture supernatant on HT-29 cells was better, reaching 96.73% after 48 h. In another in vitro experiment, *Lb. fermentum* RM28, which was found from fermented milk, could inhibit the proliferation of Caco-2 cells with an inhibition rate of 23% 47. In this study, the inhibition rates of Caco-2 were 35% and 53% when treated with *Lb. plantarum* AY01 and *Lb. fermentum* JSL8-2 culture supernatants, respectively.

Studies have shown that probiotics use the glycolysis, NF-κB or p38 MAPK pathways to exert anticancer effects 48-50. Through the analysis of transcriptome data, we found that *Lb. plantarum* AY01 may inhibit the growth of colorectal cancer cells by activating the p38 MAPK pathway. The p38 MAPK family plays an important role in complex cellular processes such as proliferation, differentiation, development, transformation, and apoptosis 51. In the protein‒protein interaction network constructed by introducing significantly different genes into STRING, many genes that were not enriched were filled between significantly different genes, forming an interaction network. Based on the criteria of incorporating interaction coefficients greater than 0.7 into subsequent analysis, we simplified the network graph generated by STRING and imported it into Cytoscape to construct a PPI with closer and more accurate interactions. In the topology analysis of the network, it is found that the network does not conform to the power distribution; that is, we cannot find a key gene connecting the whole network. After using cytoHubba for network analysis and key node mining, two core submodules could be extracted from the entire network. The differential genes in these two modules showed that *Lb. plantarum* AY01 upregulated the ubiquitination process and downregulated the cell cilia assembly process. Ubiquitination regulates various complex cellular processes, including protein degradation, protein‒protein interactions, endocytosis, cell cycle progression, and activation or deactivation of substrates 52. Therefore, any functional mutation or abnormal expression of the ubiquitination system may lead to a variety of diseases, including cancer, degenerative disease, and adaptive and innate immune-related diseases 53. In cancer, ubiquitination may lead to activation or inhibition of tumor-related pathways. In the extraction of modules involved in the ubiquitination process, most genes showed an upregulation trend, indicating that the ubiquitination process was enhanced at the transcriptional level in colorectal cancer cells during this process 54.

**Figure 12:**
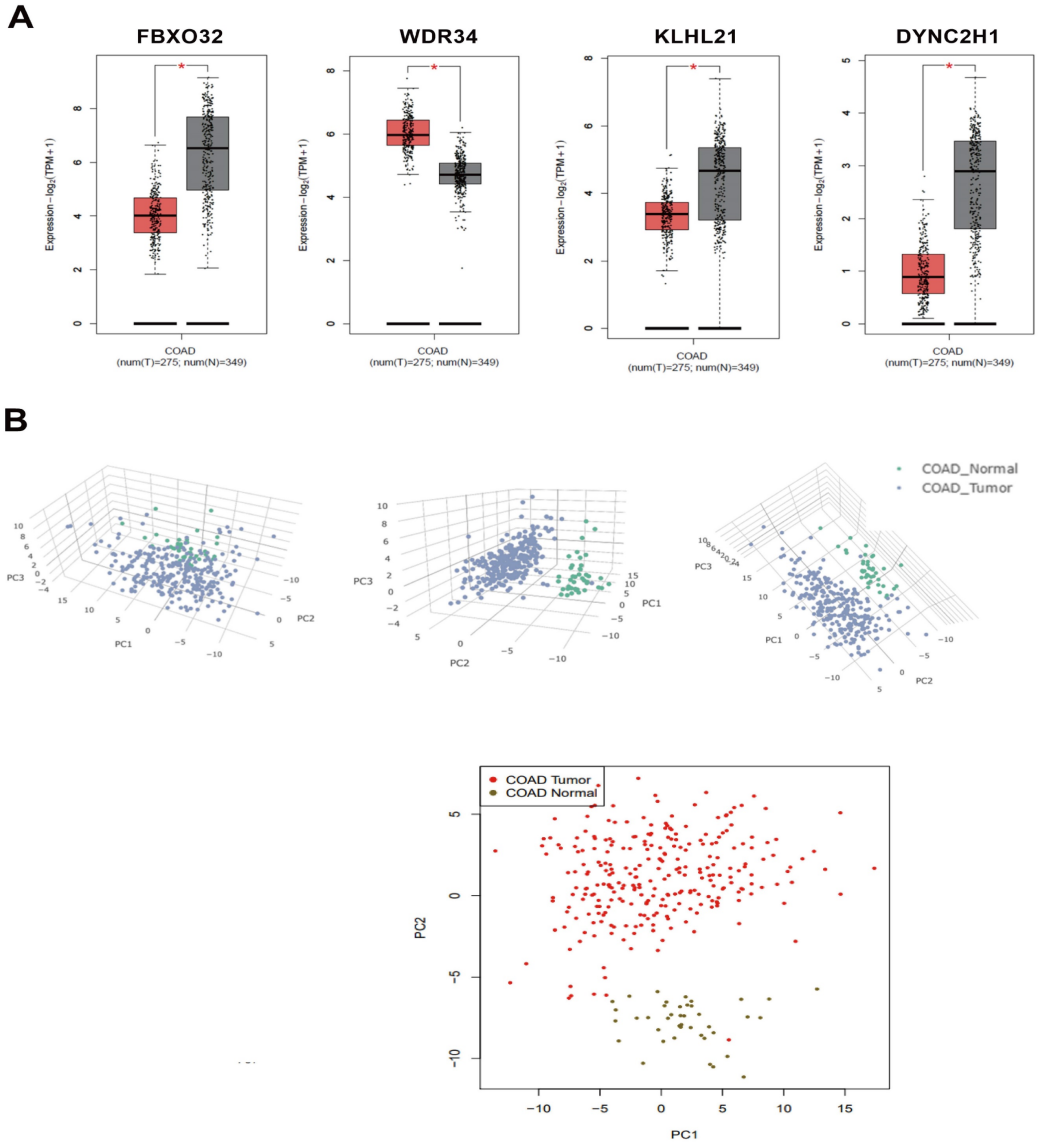
(**A**) There is a significant difference in the expression levels of *FBXO32*, *WDR34*, *KLHL21*, and *DYNC2H1* between colon cancer tissue and normal colon tissue (black represents normal colon tissue, red represents colon cancer tissue). (**B**) PCA dimensionality reduction analysis of normal colon tissue and colon cancer tissue.

These two modules are connected by the FOXO pathway, and the p38 protein in the p38 MAPK pathway can activate the FOXO pathway. The key genes of these two modules were mined in the GEPIA2 database, and four of them were found to have differences in expression between colorectal cancer patients and normal individuals. *FBXO32*, *KLHL21*, and *DYNC2H1* were significantly downregulated and *WDR34* was significantly upregulated in colorectal cancer patients. In the transcriptome, *DYNC2H1* and *WDR34* were significantly downregulated, and *FBXO32* and *KLHL21* were significantly upregulated. This showed that the treatment of colorectal cancer cells with *Lb. plantarum* AY01 can correct the expression of *FBXO32*, *KLHL21*, and *WDR34* (Figure 12). However, the p38 MAPK pathway of *Lb. plantarum* AY01 anti-colorectal cancer cells was only speculated by bioinformatics analysis, and further experimental confirmation is needed. At the same time, the three genes mentioned above may be the main target genes activated by *Lb. plantarum* AY01 in the Figureht against colorectal cancer, and further gene suppression or overexpression is needed to confirm this speculation.

Anti-colorectal cancer substances from *Lb. plantarum* AY01 were isolated and purified in this study. According to the LC‒MS results, there are still many compounds present in the final separated components. When comparing compounds in the database using their molecular weights, we discovered the substance 2'-deoxyinosine. Further confirmed by HPLC, it was preliminarily determined that 2'-deoxyinosine is the main anti-colorectal cancer substance of *Lb. plantarum* AY01. Previous studies have shown that 2'-deoxyinosine is produced by macrophages in the body and may play an important role in inflammation-driven carcinogenesis 55. After modifying the four-stranded oligonucleotide with 2'-deoxyinosine as an additional group, the obtained compound enhanced the inhibitory effect of the four-stranded oligonucleotide on the growth of tumor cells and induced S-phase arrest in tumor cells 56. But no research has shown that 2'-deoxyinosine can induce apoptosis in colorectal cancer cells. When used in combination with 5-fluorouracil, 2'-deoxyinosine can enhance the sensitivity of colorectal cancer cells to 5-fluorouracil 57. This study found that *Lb. plantarum* AY01 supernatant can inhibit colorectal cancer cells. There may be other substances in the supernatant of *Lb. plantarum* AY01 that combine with 2'-deoxyinosine to inhibit colorectal cancer cells.

This study identified the main substances and cellular pathways of *Lb. plantarum* AY01 in the treatment of colorectal cancer. New possibilities have been proposed in this study for the treatment and medication of colorectal cancer patients in the future.

## Conclusions

Overall, most of the ten strains of lactic acid bacteria in this study had certain anti-colorectal cancer capabilities, with *Lb. plantarum* AY01 having the best anti-colorectal cancer effect. The optimal conditions for *Lb. plantarum* AY01 to inhibit colorectal cancer cells were at a concentration of 64 and time of 48 h. The anti-colorectal cancer effects of *Lb. plantarum* AY01 may involve the p38 MAPK pathway. Currently, it is widely believed that the anticancer substances that probiotics such as lactic acid bacteria exert are mainly secondary metabolites such as butyric acid. The main anti-colorectal cancer substance of *Lb. plantarum* AY01 is 2'-deoxyinosine. These studies provide a favorable basis for further research on the anti-colorectal cancer effect of lactic acid bacteria.

## Figures and Tables

**Table 1:** Source of strain

Source of strain	Source
*Lb. plantarum* QB3-1	fermented soya bean
*Lb. plantarum* QB3-3	fermented soya bean
*Lb. plantarum* YM4-3	fermented soya bean
*Lb. fermentum* JSL8-2	fermented soya bean
*Lb. plantarum* AY01	fermented goat milk cheese
*Lb. latics* BZ06	fermented goat milk cheese
*Lb. casei* AS02	fermented goat milk cheese
*Lb. brevis* 3 M	fermented bean curd
*Lb. curvatus* 6 M	fermented bean curd
*P. pentosus* 2-4 L	sauerkraut

**Table 2:** Mobile phase conditions of LC‒MS

Time(min)	C_2_H_3_N (%)	H_2_O(%)	Flow velocity(mL/min)
0	20	80	1
15	22	78	1
30	45	55	1
35	55	45	1
40	55	45	1
50	100	0	1
60	100	0	1

**Table 3:** HPLC gradient

Time interval (min)	Gradient	Flow velocity(mL/min)
0-34	1%(v/v) MeOH	0.8
34-40	1-30%(v/v) MeOH	0.8
40-48	30%(v/v) MeOH	0.8
48-58	30%-1%(v/v) MeOH	0.8

**Table 4:** Top 10 KEGG pathways enriched by DAVID

KEGG pathway	Gene number	The proportion of enriched genes in total genes (%)	P value
FoxO signaling pathway	24	0.015249423	P < 0.01
Chagas disease (American trypanosomiasis)	16	0.010166282	P < 0.01
MAPK signaling pathway	28	0.017790994	P < 0.01
p53 signaling pathway	12	0.007624712	P < 0.01
Insulin signaling pathway	17	0.010801675	P < 0.01
Type II diabetes mellitus	9	0.005718534	P < 0.01
TNF signaling pathway	14	0.008895497	P < 0.01
Proximal tubule bicarbonate reclamation	6	0.003812356	P < 0.01
Renal cell carcinoma	10	0.006353926	P < 0.01
NOD-like receptor signaling pathway	9	0.005718534	P < 0.01

**Table 5:** Top 10 pathways of KEGG enriched by KOBAS

KEGG pathway	Gene number	Number of background genes	P value
FoxO signaling pathway	24	134	P < 0.01
Metabolic pathways	77	1243	P < 0.01
MAPK signaling pathway	28	255	P < 0.01
Pathways in cancer	32	397	P < 0.01
Chagas disease (American trypanosomiasis)	16	104	P < 0.01
p53 signaling pathway	13	69	P < 0.01
Insulin signaling pathway	17	139	P < 0.01
AGE-RAGE signaling pathway in diabetic complications	14	101	P < 0.01
AMPK signaling pathway	15	125	P < 0.01
cAMP signaling pathway	19	199	P < 0.01

**Table 6:** Potentially related compounds based on their molecular weight

Name	Toxicity	Role
(R)-Pantetheine	high	The synthetic precursor of CoA plays a crucial role in metabolism
(-2-Hydroxy-3-(3-indol yl) propanoic acid	high	/
(3R,5Z) 3-Hydroxy-5-dod ecenoic acid	low	/
(1R,2S)-2-Hexylcyclopro panedecanoic acid	middle	Lipid composition of *Lactobacillus*
2'-Deoxyinosine	high	Inhibits the growth of human cancer cell lines and is related to the lack of Purine nucleoside phosphorylase (PNP)
Pyroglutaminylphenylala nine	/	/
Pyroglutaminylisoleucine	/	/
Pyroglutaminylleucine	/	/
